# Supplementary material for: A computational model of altered neuronal activity in altered gravity
Source: NPJ Microgravity. 2026 Aug 7;12:70. doi: 10.1038/s41526-026-00644-7 (PMC13451429; doi:10.1038/s41526-026-00644-7)
Supplement: Supplementary file 1 [file 41526_2026_644_MOESM1_ESM.pdf]

5 Supplementary material

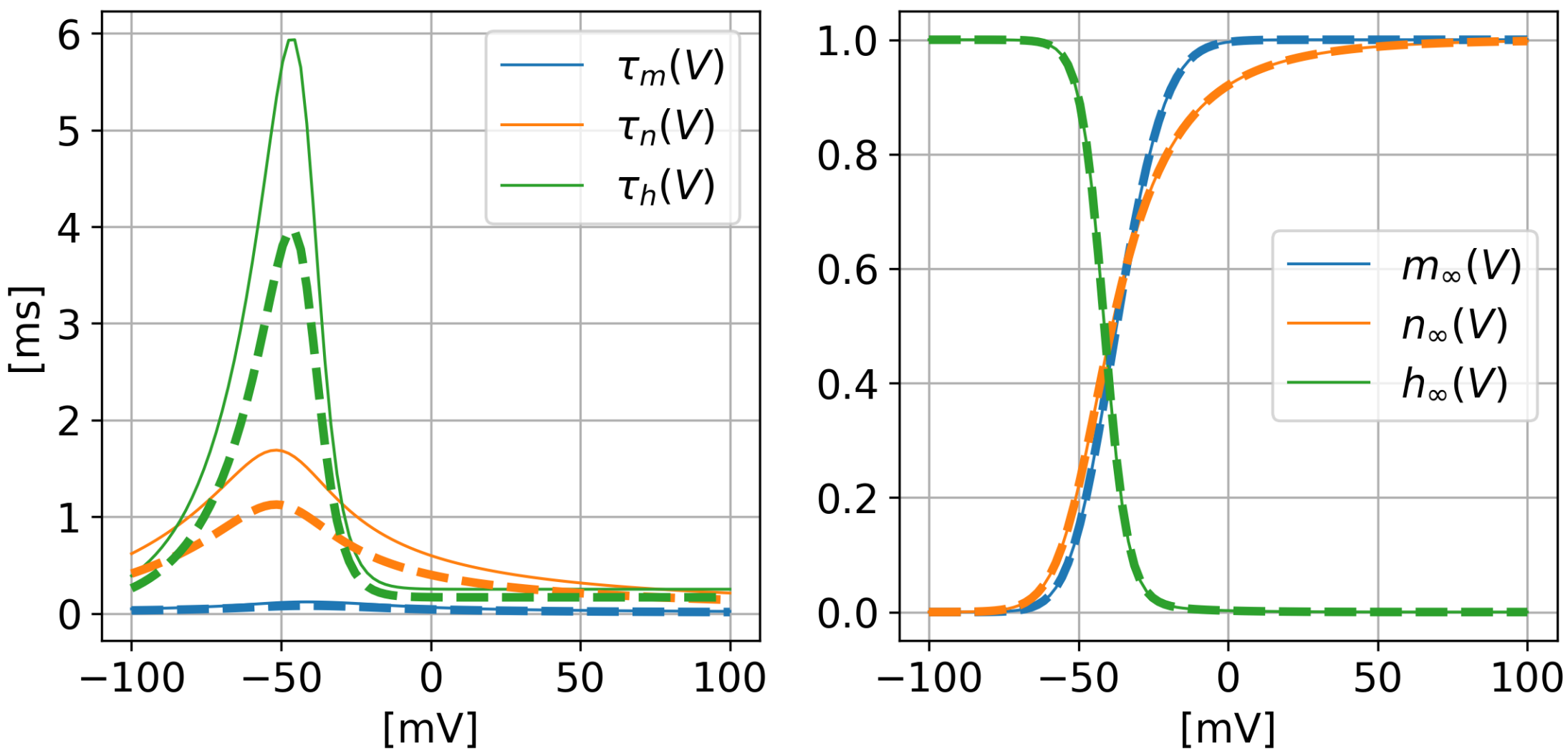

Figure S 1: Effect of λ on the gating time constants (left) and steady-state levels (right) in the Hodgkin-Huxley model. Solid line: $\lambda = 1$. Dashed line: $\lambda = 1.5$.

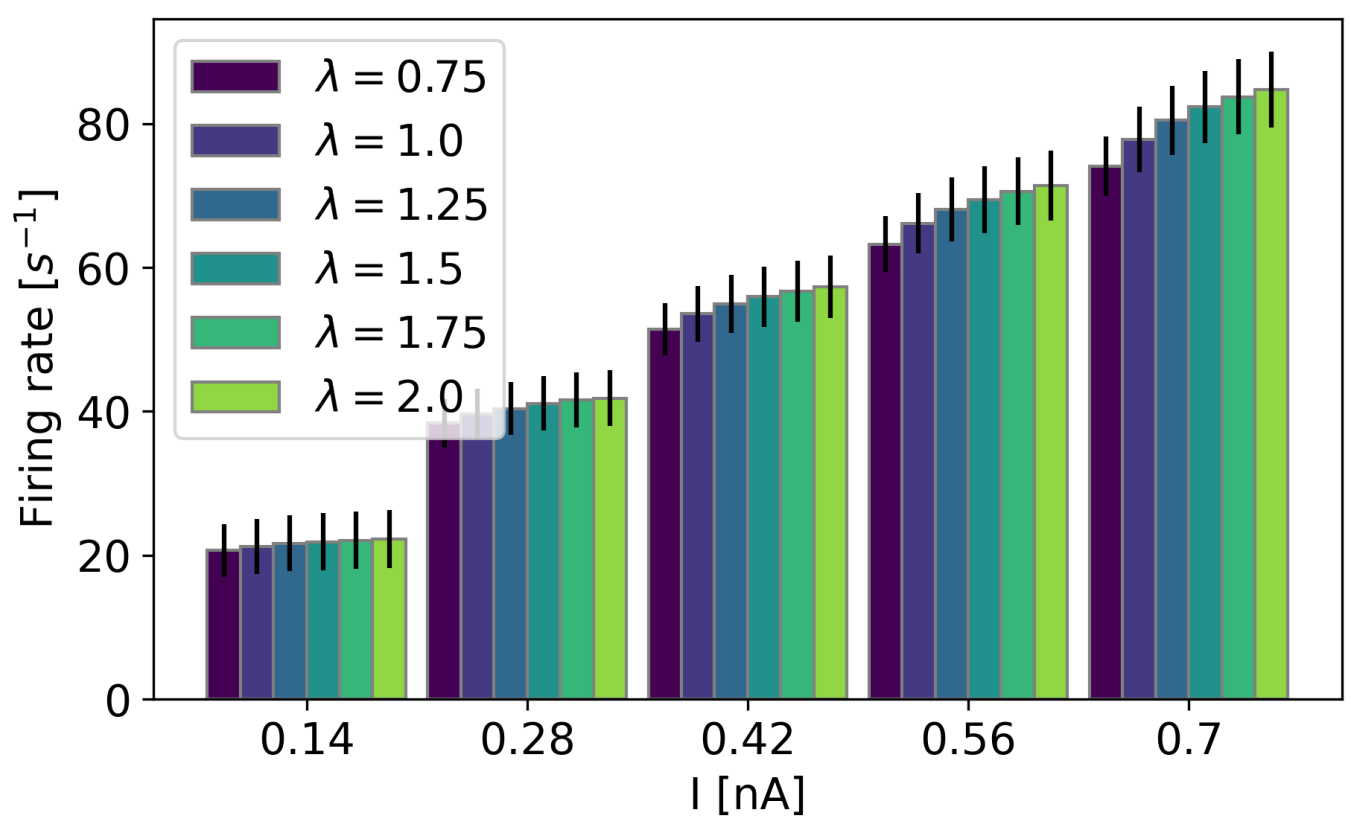

Figure S 2: Robustness analysis: same results as in Fig. 1A, with results averaged over 100 independent repetitions. For each repetition, the value of each simulation parameter was randomly drawn from a uniform distribution with bounds ±10% of the nominal values listed in Table 1. Vertical bars: s.e.m.

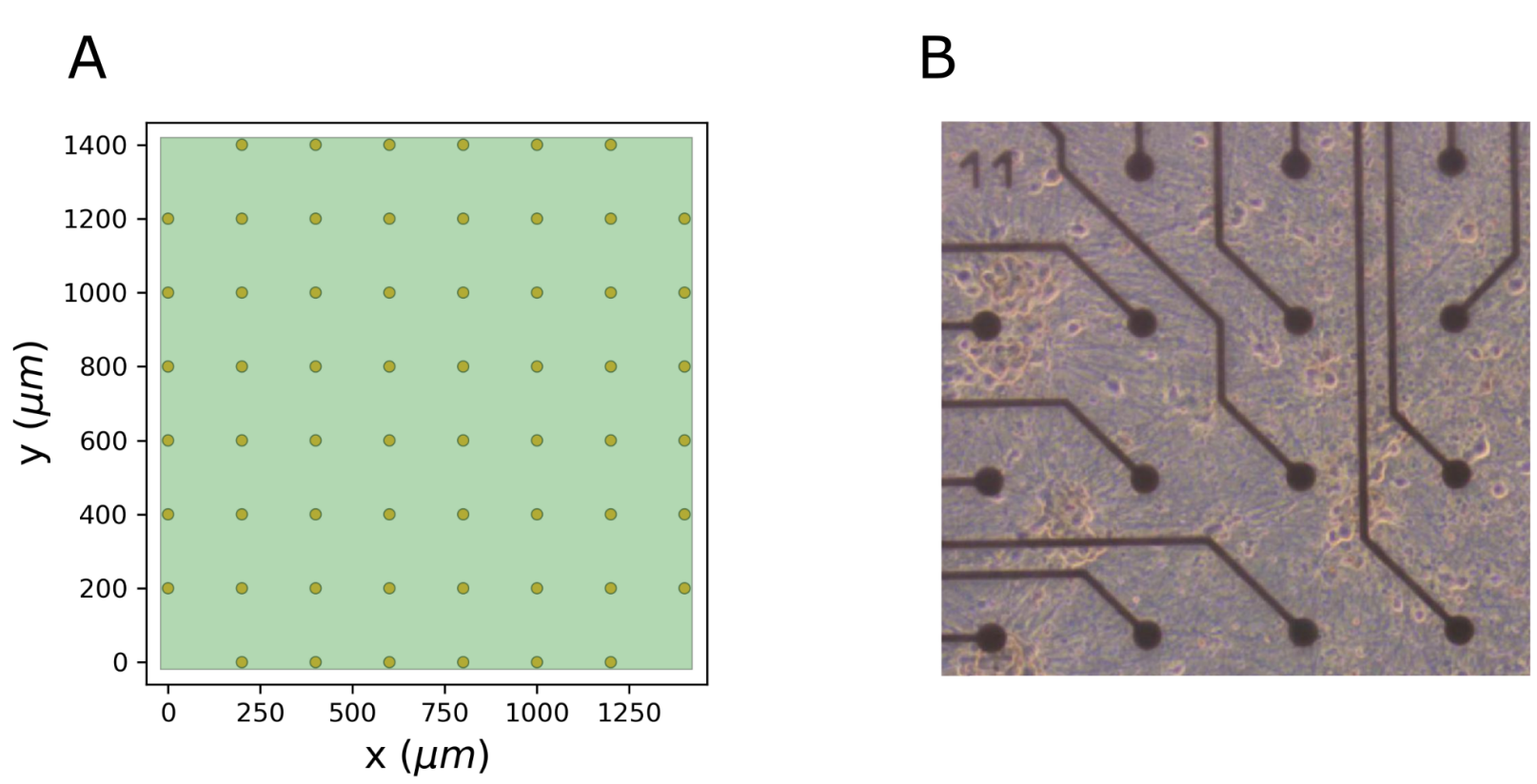

Figure S 3: A: Layout of the recording electrodes. Population activity was recorded using Multi-Electrode Array (MEA) chips featuring 60 individual electrodes (60MEA200/30iR-ITO chips, MultiChannel Systems, Germany). The 60 electrodes have a diameter of 30μm and are separated by 200μm. The extracellular voltage recorded by each electrode is sampled at 25 kHz. Each neuronal population was recorded using 2 sets of MEA chips simultaneously. B: Zoomed phase contrast microscopy image of an exemplary MEA chip showing different neurons. Reproduced from [21].
